# Long-term results of immediate implantation in the maxillary molar area with simultaneous sinus floor elevation by the crestal approach and early loading protocol: A retrospective case series follow-up study

**DOI:** 10.15171/japid.2018.011

**Published:** 2018-12-25

**Authors:** Nader Ayubian Markazi, Nasrin Akhondi, Mostafa Montazeri

**Affiliations:** ^1^Department of Periodontics, Implant Research Center, Faculty of Dentistry, Tehran Medical Sciences, Islamic Azad University, Tehran, Iran; ^2^Department of Mathematics, Islamic Azad University of Tehran, South Tehran Branch, Tehran, Iran; ^3^Periodontist, Private Practice, Shiraz, Iran

**Keywords:** Sinus floor augmentation, implantation, molar, survival rate, osteotome technique, fresh socket, bone graft, immediate placement, case-report/series

## Abstract

**Background:**

This study aimed to determine the long-term survival rate of implants placed in fresh sockets of extracted maxillary molars with simultaneous sinus floor elevation and early loading protocol.

**Methods:**

Nineteen maxillary molar teeth were extracted by tooth sectioning, and the sockets were debrided. Drilling for implant placement (Either Xive, Dentsply or Axiom, Antogyr) was terminated 1 mm short of the sinus floor with a pilot drill. Then, according to Summers’ technique, elevation of the Schneiderian membrane and bone grafting were performed. The implants were placed according to non-submerged procedure after sinus grafting and preparation of the desired osteotomy site.

**Results:**

The implants had been in function up to 5 years and the mean time of loading was 33.12 months. Analysis of crestal bone loss records indicated a mean of -0.054±0.56 mm of bone resorption (with a range of –0.86 to +0.90 mm). The amount of crestal bone resorption on the mesial and distal surfaces of implants was -0.02±0.559 mm and -0.09±0.59 mm, respectively (P=0.232). Survival rates and success rates were 100% and 95.45%, respectively.

**Conclusion:**

Immediate implant placement in the posterior maxilla with simultaneous sinus floor augmentation and early loading was a reliable and predictable approach.

## Introduction

Immediate implantation has been reported to be an acceptable treatment modality and has shown that its success rate is comparable with implant placement in healed ridges.^1^ The findings of many studies based on the histology of wound healing in the sockets provided the idea of immediate implantation into sockets of extracted teeth. Numerous studies now indicate that the immediate implantation is as successful as delayed implant placement provided that, implants have adequate primary stability.^2-6^ Immediate implant placement in the posterior maxilla has its unique features, represents specific challenges, and requires careful consideration regarding accurate positioning of the implant relative to the socket and maxillary sinus anatomy. From a restorative point of view, the implant should be placed in the interradicular bone. Based on this concept, the interradicular bone should have adequate height and width to attain sufficient primary stability.^7^ The inadequacy of the alveolar bone in the posterior maxilla following tooth extraction and immediate implant placement, in some cases, necessitates a surgical approach to overcome this problem. One option in this situation is the elevation of the Schneiderian membrane and bone grafting with various bone graft materials and allowing the socket to heal, followed by implant placement after three to four months when new bone formation and bone healing is completed. When immediate implant placement is planned in the posterior maxillary teeth that have proximity to the sinus floor, there is not sufficient height of bone in the apical area of the extracted tooth, and most of the time sinus bone grafting without implant placement is recommended. Summers’ osteotome technique^8^ is a less invasive approach for sinus grafting that can be applied after tooth extraction and in specific cases even simultaneously with immediate implantation. Kolhatkar et al^9^ described five cases with a combination of immediate implantation and sinus bone grafting by osteotome technique in maxillary premolar teeth and reported 100% survival rate during a 6- to 12-month follow-up.

The advantages of the technique above include fewer surgical interventions, elimination of the healing period, cost reduction, as well as prevention or reduction of alveolar bone loss following tooth extraction.^2^ To overcome these problems and notably shorten the treatment period and minimize soft and hard tissue relapse, this study aims to describe an approach to extract multirooted posterior teeth of the maxilla and immediately place implants with simultaneous maxillary sinus floor augmentation with early loading. This combination can dramatically reduce treatment time and lower overall cost, as well as increase preservation of bone height and width in the alveolar bone following posterior maxillary tooth extraction.

## Methods

### Surgical Procedure

Prophylactic antibiotic (1 gr of amoxicillin) was prescribed for all the patients 1 hour before surgery. Afterward, local anesthesia (4% Articaine, 1:100,000 epinephrine) was administered. The teeth were extracted for periodontal reasons, endodontic failure, severe caries, and tooth fracture. Panoramic radiography or cone-beam computed tomography (CBCT) scans were obtained preoperatively. An implant was considered as failed when one of the following criteria was detected: early or late implant mobility or extensive marginal bone loss. If not, the implant was deemed to be successful. In all the patients, healing was uneventful. The teeth were extracted as gently as possible by tooth sectioning via Piezosurgery (EMS), and the sockets were debrided prudently. Following tooth extraction, adequate bone height (5‒6 mm) of interradicular bone was a prerequisite for placing implants in an ideal restorative position with good primary stability. Using a round drill, a depression was prepared on the center of the bone septum between the roots of multi-rooted teeth, and then drilling ended 1 mm short of the sinus floor with a pilot drill.^7^ Then, according to Summers’ technique (BAOSFE)^8^, elevation of the Schneiderian membrane and bone grafting were performed. Throughout the procedure of bone-added osteotome for membrane elevation, Valsalva maneuver was conducted to ensure the integrity of the membrane. The implant was placed after sinus grafting and preparation of the desired osteotomy. The implants were either Xive system implants (Xive, DENTSPLY International) or Axiom (Axiom Antogyr, France). All the implants were positioned 1‒2 mm below the buccal and lingual plates.

**Figure 1:**
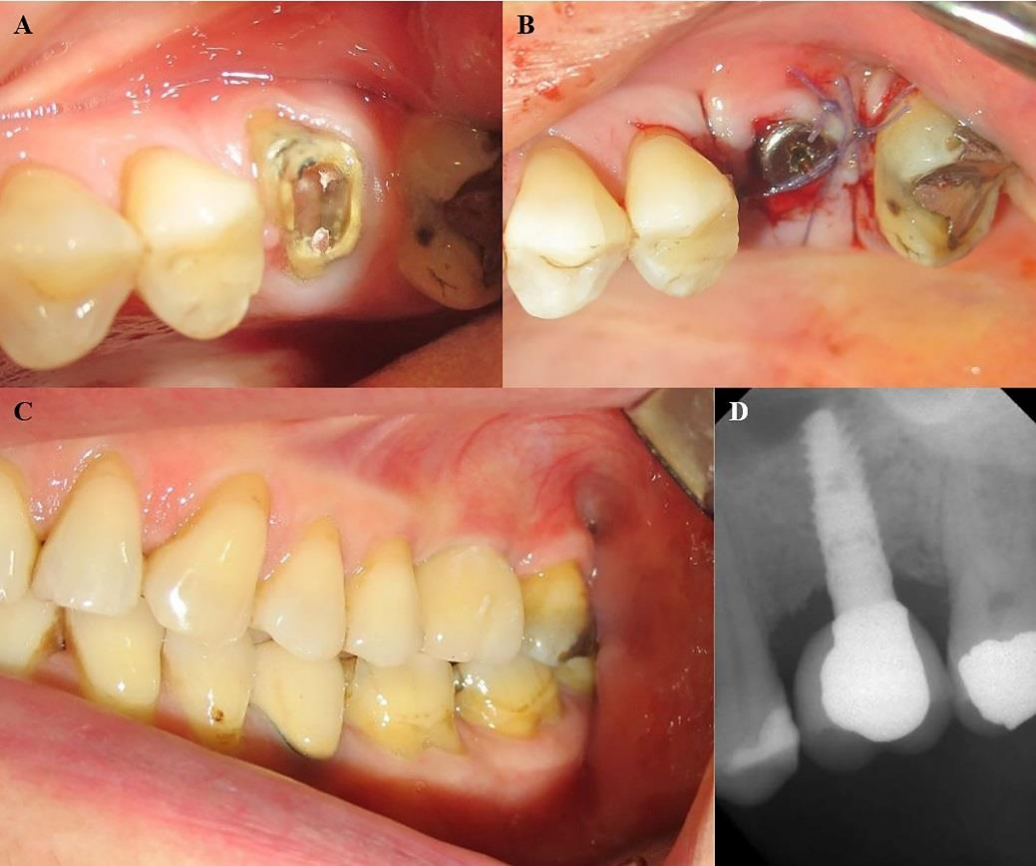
(A) Hopeless maxillary first molar which is planned to be extracted. (b) First maxillary molar extracted by piezosurgery. Xive implant immediately placed in the fresh socket. Hard and soft tissue augmentation was performed after the implant placement (Not shown). (C) The same patient as shown in Fig 1. The implant is loaded by definite restoration after 2 months. (D) Peri-apical radiograph of the implant restoration after 32 months of follow-up. No signs of pathology are seen on the radiograph.

**Figure 2:**
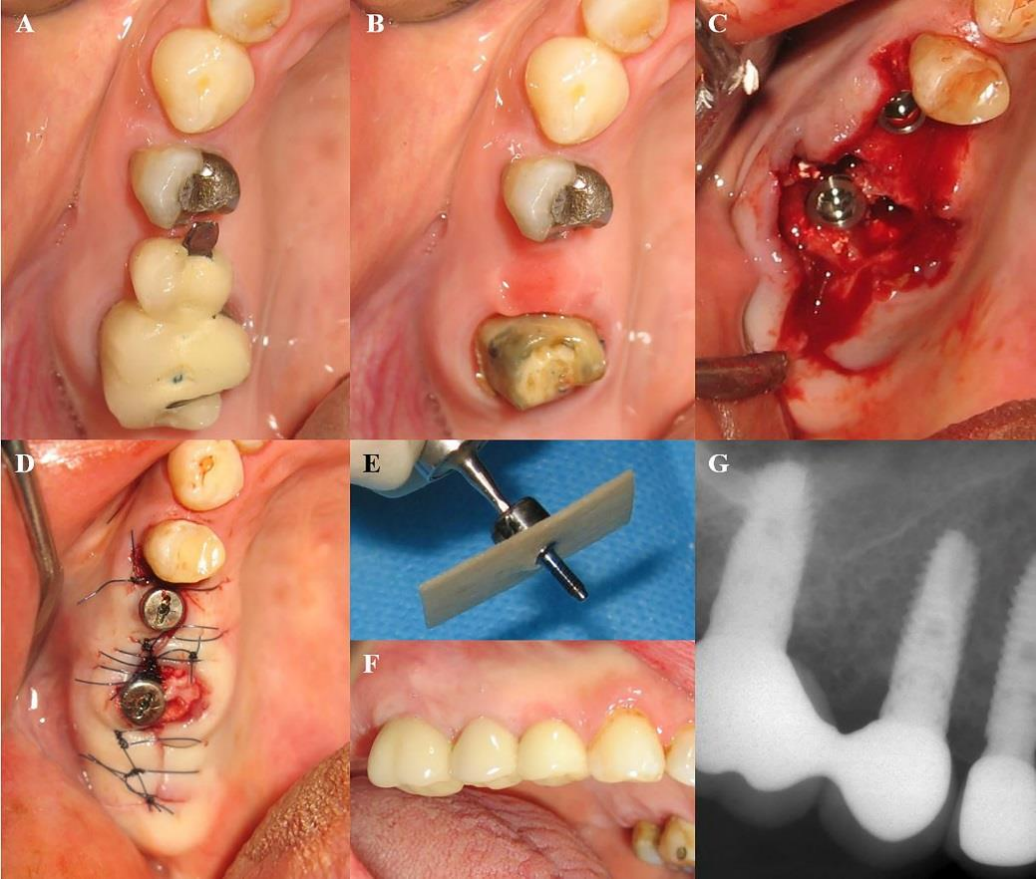
(A) A 52-year-old female patient. Restoration of maxillary second premolar and first molar presented with food impaction and the patient was uncomfortable on chewing. (B) Photograph of the site after removal of the defective fixed partial denture. (C) Following the minimally invasive extraction of hopeless teeth, two implants (Xive system) were inserted. Sinus floor elevation by crestal approach was performed before the placement of the maxillary first molar implant. (D) Flaps were sutured by 4/0 Vicryl suture. (E) The collagen membrane is secured by a healing cap. (F) Clinical view of the maxillary right quadrant with implants in function. (G) Peri-apical radiograph of inserted implants after 62 months.

In some cases, osteoplasty and osteotomy were performed for enhanced flap adaption. The geometric characteristics of implants are presented in Table 1. The root sockets were filled with bone grafting material (Cerabon or Tutodent, Tutodent Microchips Tutodent, TUTOGEN Medical GmbH) and covered by acellular dermal allograft (SureDerm, Hans Biomed Co., Ltd.) or collagen membrane (Tutopach membrane, TUTOGEN Medical GmbH). All the implants were placed according to the non-submerged technique, and the membranes were secured by connecting healing abutments. In some cases, the soft tissue sealing of the socket was obtained by a coronally advanced flap or connective tissue pedicle graft from the palate. The flaps were positioned coronally over the sockets and stabilized by single interrupted 4-0 Vicryl sutures. All the patients received postoperative medication, including prescriptions for 500 mg of amoxicillin TID for one week; Novafen (ibuprofen, 200 mg; caffeine, 40 mg; and acetaminophen, 325 mg; Alhavi Pharmaceutical Co.) QID; and a 0.2% chlorhexidine mouthwash BID. The sutures were removed two weeks later. Healing was uneventful except in one patient who had abscess formation. In this patient, implant mobility was detected, and the implant was removed, and bone grafting was performed at the defect. All the patients were asked at the 2-week follow-up visit to continue using the chlorhexidine mouthwash and to avoid brushing over the implant area for another three weeks. After a healing period of 6‒8 weeks, osseointegration was confirmed by clinical and radiographic examinations and a recording Periotest value and all the implants were loaded by delivery of definitive restorations (Cases 1‒8) (Figures 1 and 2). The geometric characteristics of implants and demographic characteristics of patients are presented in Table 1. The research protocol was conducted according to the 1975 Declaration of Helsinki, as reviewed in 2013.

**Table 1:** The geometric characteristics of implants and demographic characteristics of patients

**Patient**	**Implant**	**Follow-up(month)**	**Sex**	**Age(year)**
**1**	Xive 4.5*/11**	32	F◊	53
**2**	Xive 4.5/11	62	M◊◊	57
**3**	XIVE 4.5/9.5	55	F	44
**4**	Axiom 4.8/12	48	M	56
**5**	Xive 3.8/11	54	F	52
	Xive 3.8/9.5	54	F	52
**6**	Axiom 4/10	56	F	51
**7**	Xive 4.5/9.5	58	M	68
**8**	Axiom 4.6/10	18	M	42
**9**	Xive 4.5/9.5	21	F	67
	Xive 3.8/13	21	F	67
**10**	Xive 4.5/13	34	F	49
**11**	Xive 4.5/11	12	M	74
**12**	Xive 4.5/9.5	24	F	61
**13**	Xive 3.8/11	38	F	63
	Xive 4.5/11	38	F	63
**14**	Xive 4.5/11	26	F	48
**15**	XIVE 4.5 /11	24	F	65
**16**	Xive 4.5/11	36	F	52
**17**	XIVE 3.8/11	10	F	50
**18**	XIVE 34.5/11	13	M	38
**19**	XIVE 4.5/11	32	F	67

*Diameter, ** Length, ◊ Female, ◊◊ Male

### Radiographic Evaluation

The patients were re-evaluated 1‒8 years after implant placement (mean follow-up period was 33.12±16.98 months). An implant was considered as failed when one of the following was detected: early or late implant mobility or extensive marginal bone loss. Otherwise, an implant was deemed as successful. Periapical radiographs were obtained using the MyRay Digital Imaging X-ray sensor and an X-Pod Wireless Digital System (Cefla SC Dental Group, Italy). The distance between the first bone‒implant contact (FBIC) and implant shoulder on mesial and distal sides of implants was measured. The linear measurements were calibrated by monitoring the actual length of implants by the X-Pod system. The assessed outcomes were: implant survival rate, implant success rate, and crestal bone resorption.

### Implant Success Criteria

Implant success was defined consistent with the Buser criteria^10^ as follows: 1) lack of implant mobility; 2) absence of pain; 3) absence of infection in peri-implant tissues; and 4) lack of radiolucency around the implant at the time of examination. According to a systematic review, the most common criteria for evaluating the success of implants was the absence of mobility, pain, radiolucency and crestal bone resorption (>1.5 mm).^11^

### Statistical Analysis

For statistical analysis and determining implant survival, implant success and crestal bone loss the implant was used as the statistical unit. Statistical measures (mean, SD, minimum, maximum, range) were calculated for quantitative variables, and distribution normality assumptions were assessed by the Kolmogorov–Smirnov test (P>0.05). For comparisons, independent sample t-tests were used with quantitative variables between the groups being defined by sex; a paired‑samples t-test was used for comparisons between bone loss in the mesial and distal aspects. In addition, a 95% confidence interval (CI) for a mean of quantitative variables was calculated. The level of statistical significance was set at P≤0.05. For crestal bone resorption, means and standard deviations were calculated. Statistical comparisons were performed by t-test, using SPSS.

## Results

The data related to 19 patients who had received immediate implant therapy with simultaneously closed maxillary sinus graft and early loading were used to determine the survival rate of implants and mean crestal bone loss. The recorded data were related to 19 patients (12 females and 7 males) with a mean age of 56.28 years. All the patients had received an implant at least one year before the study. The mean follow-up assessment period was 33.12±17.01 months (Table 2). Peri-implantitis and pocket formation was detected in one patient 15 months after implant placement due to a sizeable marginal gap and remnants of temporary cement. This case was treated according to principles of guided bone regeneration (GBR) after which a new crown was fabricated. At the time of re-evaluation (16-month follow-up), bone regeneration was evident along with the elimination of the pocket (case #7). No implant had been lost in function, yielding a cumulative survival rate of 100%. The implants had been in use with a mean time in use of 35.27 ±17.01 months.

**Table 2:** Mean follow-up period of the patients

		**Female**	**Male**	**Total**
**Follow-up time (month)**	**Mean**	33.70	35.17	35.27
	**SD**	14.02	23.36	17.01
	**Minimum**	10.00	12.00	10.00
	**Maximum**	56.00	62.00	62.00
	**Range**	46.00	50.00	52.00

P=0.893* *NSP (Based on independent-samples t-test)

Analysis of crestal bone loss records indicated a mean of -0.054±0.56 mm bone level. Mean bone levels on mesial and distal surfaces of implants were -0.02±0.55 mm and -0.09±0.59 mm, respectively (P=0.232, Tables 3 and 4). There was a significant difference between genders, regarding the total amount of marginal bone loss (Table 3). In most cases, crestal bone resorption was limited to the collar of implants, except one with peri-implantitis, in which bone resorption was extended to the threads of implants. Mean bone level for each implant, for purposes of this study, was calculated by summing up of bone gain and bone loss at different surfaces of the implant. In the present study, 45.5% of implant surfaces (mesial and distal) exhibited bone loss; 22.7% of implant surfaces showed no bone loss and 31.8% of implant surfaces had bone levels coronal to the reference point (implant platform). At the time of evaluation, all the implants were present, and pain, radiolucency and crestal bone resorption >1.5 mm were found only in one implant. Therefore, based on considered implant success criteria, the success rate was 95.45%.

**Table 3:** Means of the total crestal bone level of the implants

		**Sex**		
		**Female**	**Male**	**Total**
**Total bone level (mm)**	**Mean**	–0.13	0.29	–0.054
	**SD**	0.58	0.41	0.56
	**Minimum**	–0.98	–0.34	–0.98
	**Maximum**	+0.80	+0.92	+0.92
	**Range**	1.78	1.26	1.9
**95% CI**	(–0.37, 0.10)	(0.03, 0.55)	(0.23, 0.12)
**P -value**	0.029***		

***significant (based on independent-samples t-test)

**Table 4:** Mean bone level on the mesial and distal surfaces of implants

		**Female**	**Male**	**Total**	**95% CI for total mean**	**P-value**
**Mesial Bone Level** **(mm)**	**Mean**	–0.13	+0.37	–0.02	(–0.26, 0.23)	0.556**
	**SD**	0.57	0.35	0.55		
	**Minimum**	–0.76	0.00	–0.76		
	**Maximum**	+0.80	+0.92	+0.92		
	**Range**	1.56	0.92	1.68		
**P-value**		0.07*				
Distal Bone Level* **(mm)**	**Mean**	–0.14	+0.21	–0.09	(–0.36, 0.18)	
	**SD**	0.61	+0.48	0.59		
	**Minimum**	–0.98	–0.34	–0.98		
	**Maximum**	+0.80	+0.88	+0.88		
	**Range**	1.78	1.22	1.86		
**P-value**		0.232*				

*NS (based on independent-samples t-test) ** NS (based on paired-samples t-test)

## Discussion

The results of this retrospective clinical study showed that immediate implantation in the posterior maxilla with simultaneous sinus bone grafting and early loading has a high survival rate. Several human clinical studies have demonstrated that the immediate implantation has results comparable with delayed implant placement regarding survival rate (94–100%).^12-13^ Langet al^14^ reported a two-year survival rate of 98.4% based on a systematic review. In the present study, the cumulative survival rate was 100% similar to the reported rate in the studies above.^12-14^ According to another systematic review by Kinaia et al,^15^ the amount of crestal bone resorption around immediately placed implants was less than that in healed sockets. Espositoet al^16^ stated that the amount of crestal bone resorption for immediately and delayed placed implants, one year after being in function, was 0.23 mm and 0.29 mm, respectively, and reported that the difference was statistically significant. The mean bone loss in this study was -0.054±0.56 mm. One reason for less bone loss with immediate implant placement might be the subcrestal position of the implant that compensates for bone loss following the bone remodeling that occurs after loading. The other reason might be the use of platform switching concept in selecting abutments for definite restorations.

Achieving good primary stability is the cornerstone of the standard protocol for the implant placement and is a prerequisite for osseointegration. This stability can be accomplished by placing implants longer than the apical portion of the extraction socket.

The immediate implantation in the maxillary molar area is highly patient- and site-specific. The first element that must be determined is the required minimum implant dimensions for a specific patient’s treatment plan. Once these parameters have been established, the clinician should decide whether or not the successful placement of the implant of the appropriate dimensions is feasible. Other factors that affect the decision about immediate implant placement are the characteristics of the tooth to be replaced and the morphology of tooth socket, especially interradicular bone. The interradicular bone must be maintained whenever possible. From a prosthetic point of view, the implant should be placed in the interradicular bone. A study showed that the mean distance from sinus floor to maxillary first molar furcation was 6.51 mm in CBCT examination, which is long enough to deliver good primary stability.^17^ Tooth sectioning and use of piezosurgery and a periotome enhance the preservation of the interradicular bone that is fundamental for this procedure. Most of the time, implant placement is dictated by the morphology of the residual ridge, and this might be a more prominent concern in cases of immediate implantation in the socket of multirooted maxillary teeth. The simplicity of placing the implant in the root socket might persuade the clinician to perform such an approach. Although this method might lead to osseointegration of implants, it is not suggested due to the long-term problems concerning difficulty in plaque control and uncontrolled non-axial forces placed on the implant during the daily function. As mentioned previously, the placement of the implant after tooth extraction is highly patient- and site-specific. Therefore, the clinician must recognize situations in which such a treatment modality is not possible in a predictable manner. Patients should be informed about this probability before initiation of treatment. Failure to communicate clearly will result in misunderstandings, jeopardizing both the results of treatment and patient satisfaction.

The additional phase of this study was implementing early loading protocol. Early implant loading might lead to implant failure because of the encapsulation of the implant by connective tissue and the lack of osseointegration^18^. However, recent studies have indicated that early loading can be an acceptable protocol and be accompanied by implant success comparable with a delayed-loading protocol.^19,20^ Two main factors for achieving osseointegration are the primary stability of the implant and a lack of micromovement during the healing phase. According to conventional loading protocol, an implant should not be loaded for 3 to 6 months after placement.^18^ Shortening the healing time might be beneficial to patients. This is why the concept of immediate and early loading has been proposed by some authors. Kreja et al^21^ reported that loading during the healing phase of implantation is not completely detrimental to osseointegration and loading at suitable amounts may promote osteogenesis. Immediate and early loading of implants is considered as the approved protocol in the case of decent bone quality and ideal locations.^22^ In a prospective clinical study, the outcomes of early loading of delayed and immediately placed implants were compared, and the authors reported that the short-term results of both loading protocols were comparable for implants placed in the mandibular anterior region.^23^ A retrospective clinical study regarding the immediate loading of implants placed in fresh sockets of molar teeth stated that this protocol could be a feasible alternative for molar replacement with dental implants.^24^ To overcome problems related to insufficiency of bone height on the posterior portion of the maxilla, sinus bone grafting was introduced by Tatum^25^ and published by Boyen and James in 1980.^26^ Summers^27^ introduced a technique named, “Osteotome sinus floor elevation (OSFE)” in 1994. In this approach, specifically designed osteotoms with increasing diameters are used to fracture the sinus floor and elevate the Schneiderian membrane. Summers^8^ later introduced “the bone-added osteotome sinus floor elevation” (BAOSFE) to enhance the approach above. In case of the presence of at least 5 mm of residual bone, the BAOSFE approach is used as a less aggressive technique compared with the open sinus bone graft to increase bone height in the posterior maxilla. In this situation, implants can be placed simultaneously. Pjtursson et al^28^ demonstrated the efficacy of the osteotome technique for maxillary sinus bone grafting. These authors showed that when using bone graft materials, the height of bone dramatically increased as evident by radiographic evaluation after healing. That study reported that the survival rate of implants placed by osteotome technique, 3.2 years after loading was 97.4%. According to these authors, this technique is a reliable method for replacing missing teeth by implants in the maxillary premolar and molar area, provided that enough residual bone height is available (5 mm) and the lower border of sinus cavity is flat. Recent studies have shown that the defect around the implant following tooth extraction and immediate implantation can be healed without any intervention such as bone grafting and guided bone regeneration procedures. These defects frequently are four-walled, without fenestration and dehiscence. It has been shown that when the size of the peri-implant defect is <2 mm bone grafting or guided bone regeneration is not necessary.^13,29^ However a recent study by Tarnow et al^30^ strongly recommended bone grafting the peri-implant defect irrespective of the size of the defect. Sanz et al^31^ evaluated the advantage of using bone grafting in combination with immediate implant placement. They stated that placing bone graft materials in the defect between the implant and the buccal bone plate of socket significantly reduced horizontal bone resorption in the buccal bone after immediate implant placement. In this study, the peri-implant defect after immediate implantation was grafted with graft material. It has been stated that the horizontal component of a peri-implant defect following immediate implantation is the most significant factor in the determination of final bone-implant contact.^32^ It has been stated that in the case of immediate implantation, the large marginal gap should be filled with various bone grafting materials and the membrane might be applied. According to these authors, this approach would prevent soft tissue collapse, which is a frequent and unwilling consequence of tooth extraction.^33,34^ In this study all the implants were placed with healing abutments and allowed to heal according to one stage protocol. The one-stage approach avoids the second surgery for exposing and connecting healing abutments and reduces treatment time. Many clinical studies compared one- and two-stage implant placement with GBR. According to these studies, a non-submerged protocol can lead to successful bone regeneration in peri-implant defects.^35-38^ However, one study recommended a two-stage approach in the case of GBR.^35^ A study by Juodzbalys^39^ showed that non-submerged protocol with simultaneously guided bone regeneration could lead to predictable treatment outcomes after five years.

The present study had a retrospective design. Consequently, there might be a bias in the selection of patients because some information about patients might be omitted during data collection. Prospective controlled clinical trials, including larger samples, are recommended to verify the reliability of proposed treatment modality.

Immediate implant placement in the maxillary molar teeth with simultaneous maxillary sinus floor augmentation and early loading is a combination of three reliable surgical techniques that could have a high survival rate. This combined approach could be valuable for shortening the duration of implant therapy in maxillary molar teeth that are close to maxillary sinus and could improve the available bone height for placing implants with the desired length. Based on the limited number of patients and the design of the study, the results should be considered with caution, and randomized clinical trials with larger sample sizes are recommended.

## Authors’ contributions

The study was planned by NAM. Data collection was carried out by NAM and NA; statistical analyses and interpretation of data were carried out by NA. The manuscript was prepared by NAM and MM, and revised by MM. All the authors have read and approved the final manuscript for submission.

## Ethics approval

The Ethics Committee in Faculty of Dentistry, Islamic Azad University of Tehran, approved the study protocol.

## Conflict of Interests

The authors declare no financial interests regarding any materials, products or brands mentioned in this article.
